# Sociodemographic, obstetric characteristics, antenatal morbidities, and perinatal depressive symptoms: A three-wave prospective study

**DOI:** 10.1371/journal.pone.0188365

**Published:** 2018-02-08

**Authors:** Ying Lau, Tha Pyai Htun, Ho Keung Dennis Kwong

**Affiliations:** 1 Department of Alice Lee Centre for Nursing Studies, Yong Loo Lin School of Medicine, National University of Singapore, Singapore; 2 Department of Alice Lee Centre for Nursing Studies, Yong Loo Lin School of Medicine, National University of Singapore, Singapore; 3 School of Health Sciences, Macao Polytechnic Institutes, Macao Special Administrative Region of the People’s Republic of China, China; Swansea University, UNITED KINGDOM

## Abstract

**Objectives:**

This study aimed (1) to investigate the pattern of perinatal depressive symptoms, and (2) to determine the relationships between sociodemographic characteristics, obstetric factors, antenatal morbidities, postnatal conditions, and perinatal depressive symptoms using a structural equation model (SEM).

**Method:**

A three-wave prospective longitudinal design was used for 361 women in their second trimester, third trimester, and at six weeks postpartum. The Edinburgh Postnatal Depression Scale (EPDS) was used to assess the depressive symptoms.

**Results:**

The intensity of depressive symptoms was the highest in the second trimester among the three waves. The SEM showed that unmarried status, unplanned pregnancy, gestational diabetes, and headache were significantly associated with EPDS in the first and second waves. The EPDS in the first wave was able to predict the EPDS in the second and third waves. The SEM has satisfactorily fit with the data (chi-square/degree of freedom = 1.42, incremental fit index = 0.91, Tucker-Lewis index = 0.90, comparative fit index = 0.91, and root mean square error of approximation = 0.03).

**Conclusion:**

The findings highlight the significance of monitoring depressive symptoms in the second trimester. Findings from this study could be useful in the design of effective intervention among women with unmarried status, unplanned pregnancy, gestational diabetes, and headache in order to reduce risk of perinatal depressive symptoms.

## Introduction

The perinatal time is a sensitive period for a considerable incidence of depressive symptoms [1] because of profound physiological and psychosocial changes [2]. A meta-analysis reported that 23.8% of women suffer from depressive symptoms between the first trimester to one year postpartum [3]. The negative consequences of perinatal depressive symptoms are substantial and extend not only to the new mother and neonatal outcomes [4,5] but also have long-term consequences on the offspring’s behavioral and emotional problems in childhood [6] and adulthood [7]. Thus, advancement in our understanding of perinatal depressive symptoms is needed to aid reallocation of health resources through the development of a preventive approach in early identification of at-risk women and the creation and implementation of effective target interventions.

The pattern of depressive symptoms is inconsistent during the perinatal period for women in Australia [8], Canada [9], France [10], Mexico [11], and Hong Kong [12]. Depressive symptoms are less prevalent in the second trimester compared with the third trimester in Australia but more prevalent in the second trimester compared with the third trimester in Canada [9]. One study in Mexico found more depressive symptoms at six weeks postpartum compared with the third trimester [11], but another study in France [10] observed the same prevalence rate in these two periods. However, the intensity of depressive symptoms is the highest in the second trimester compared with that in the third trimester and six weeks postpartum [12]. The differences in prevalence rates of perinatal depressive symptoms across studies might be explained by various measurement instruments, different cut-off criteria and sociodemographic variances at different time points of perinatal period [13]. Clearly, antenatal depressive symptoms are significantly associated with an increased risk of postnatal depressive symptoms [14,15], but the specific time-point during pregnancy for predicting postnatal depressive symptom remains inconclusive.

Contradictory findings regarding perinatal depressive symptoms are found in literature [16,17]. Controversial factors include age [9,11], educational level [1,11], marital status [11,18], income [9,15], employment status [11,18], pregnancy intention [9,11], and parity [11,19]. The varying methodologies across different studies highlight the importance of prospectively studying the course of depressive symptoms and potential risk factors at different times over the perinatal period.

Experiencing antenatal morbidities may have a significant impact on depressive symptoms [20,21], especially to common antenatal morbidities, such as gestational diabetes (GDM), preeclampsia, headache, nausea, and vomiting [22]. However, previous studies showed that GDM [21,23], preeclampsia [22,24], headache [25,26], nausea, and vomiting [20,22] are not consistently associated with perinatal depressive symptoms. Moreover, the association between postnatal conditions and postnatal depressive symptoms has been explored, but the results for mode of delivery [27,28], gestation of infant [22,29], and role of newborn gender [28,30] are inconsistent. Presumably, mothers of infants being cared for in a neonatal intensive care unit (NICU) may have a higher risk of postnatal depressive symptoms [31] because of intensive fear or anxiety about their sick infants, but one study [32] reported that there is no association. By identifying the antenatal morbidities and postnatal conditions, health care professionals can ideally focus on early treatment and management, thereby minimizing complications and potentially lowering the odds of perinatal depressive symptoms.

Overall, prior findings on sociodemographic factors, obstetric factors, antenatal morbidities, and postnatal conditions are still heterogeneous, and factors are likely to vary by cultural context [17]. Previous research designs [10,11] have been focused on third trimester of pregnancy rather than second trimester of pregnancy that do not allow for the assessment of depressive symptoms at earlier time-point during pregnancy; how the specific factors are related to the time-points, and ultimately at what time-points health care professionals may consider intervening in an effort to deliver the most effective intervention. Further prospective evidence is needed to obtain a better understanding of the specific factors associated with the depressive symptoms at specific stages of the perinatal period.

## Methods

### Hypothetical model

**Fig 1:**
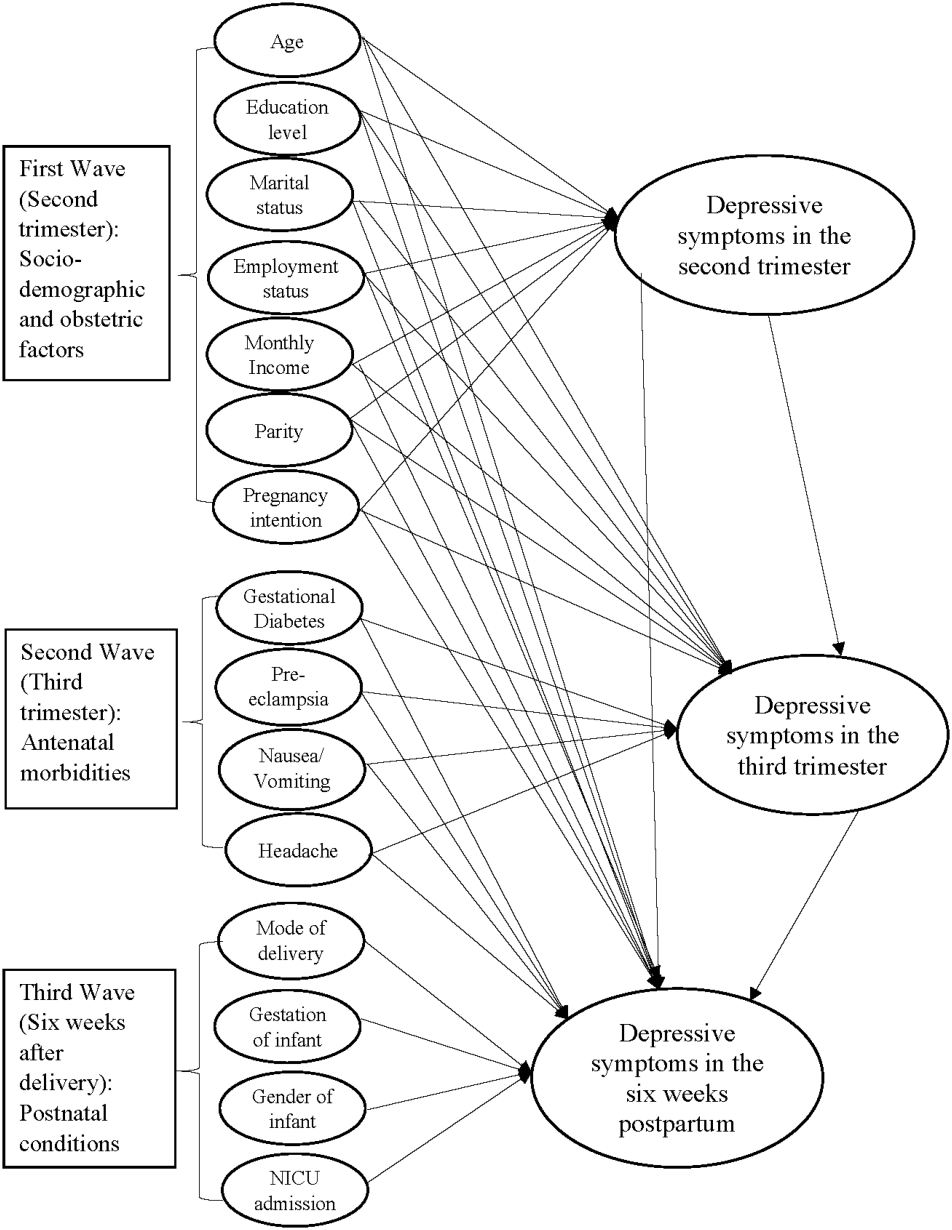
Hypothetical model of sociodemographic, obstetric factors, antenatal morbidities, and postnatal conditions on perinatal depressive symptoms in 3 waves. Solid line arrows indicate hypothetical relationships.

A hypothesized model was formulated (Fig 1) in the present study by integrating the concepts of a model of risk factors for antenatal depression [33] and a psychosocial model of antenatal depression, postnatal depression, and parenting stress [34] to understand the relationships between study variables. The hypothesized model postulates that sociodemographic factors, obstetric variables, antenatal morbidities, and postnatal conditions are associated with different time-points of perinatal depressive symptoms. Early antenatal depressive symptoms can predict late antenatal [12] and postnatal depressive symptoms, and late antenatal depressive symptoms can also predict postnatal depressive symptoms [14,15].

Only a few studies have employed structural equation modeling (SEM) to identify potential risk factors for perinatal depressive symptoms. The SEM approach is the preferred analytic strategy for identifying a complex investigation of multiple variables that affect one another simultaneously across multiple time-points by reducing bias from measurement error [35]. The approach is appropriate for a longitudinal data set to test the direction of causality between the study variables [36]. The present study aimed (1) to investigate the pattern of perinatal depressive symptoms, and (2) to determine the relationships between sociodemographic characteristics, obstetric factors, antenatal morbidities, postnatal conditions, and perinatal depressive symptoms using SEM.

### Participants and procedure

Participants in the present study were perinatal women recruited from a government hospital in Macau. Macau is a mixture of eastern and western cultures because it was a Portuguese colony from 1557 until 1999. Macau, with a land area of 30.3 km^2^, is a special administrative region of the People’s Republic of China on the southern coast of China, and its total population was estimated at 571,612 in 2015. Ethical approval that complied with the Declaration of Helsinki was obtained from the Macau Health Bureau. Non-probabilistic convenience sampling was adopted. As per the power analysis for the SEM which was used to calculate the sample size [37], the minimum required sample size is 238 participants to achieve a power of 0.80, an effect size of 0.2, and a probability level of 0.05. A sample size of 361 women was used to achieve adequate power to carry out the planned hypothesis test. The inclusion criteria included (1) being primiparas or multiparas and (2) being able to read and write Chinese. The exclusion criteria included (1) not supplying written informed consent, (2) carrying a fetus with a congenital disease, (3) having a diagnosis of depression/psychiatric problems in current pregnancy, and/or (4) having a previous history of perinatal depression or psychiatric problems.

**Fig 2:**
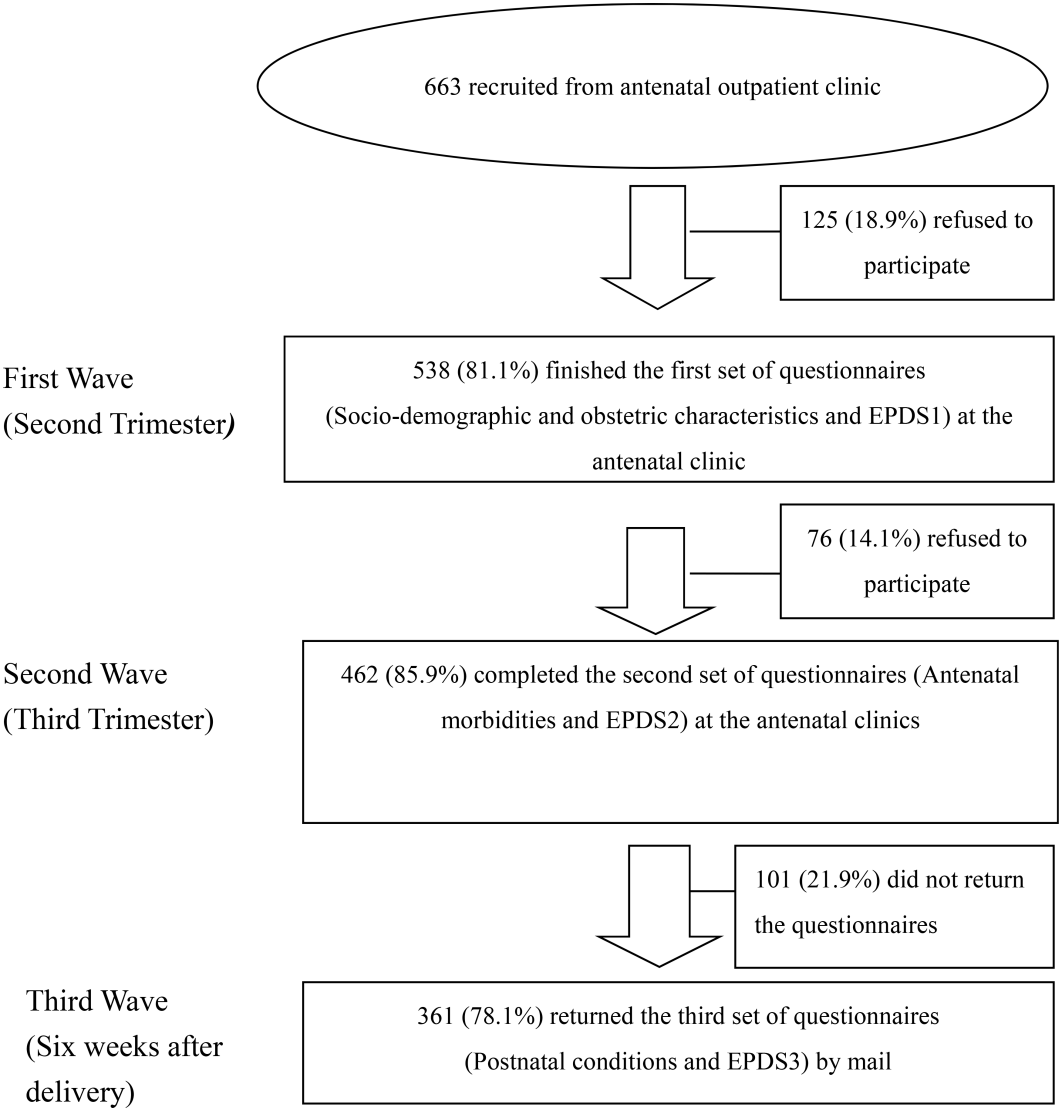
Flowchart for the implementation of the study.

An experienced research assistant determined eligible for inclusion based on their obstetric record. All eligible women who attended the antenatal outpatient clinic were invited. A full explanation of the study was given, and written consents were obtained. The participation in the study was voluntary and respondent’s confidentiality was assured. The completed questionnaires were de-identified to prevent a respondent’s identity from being connected with information. A three-wave prospective longitudinal research design was used. The women completed the first two sets of self-administrated questionnaires while waiting for a routine checkup at their antenatal clinic during their second and third trimesters. The third set of questionnaires was collected at six weeks postpartum via phone calls. Fig 2 provides a flow chart of data collection for three waves of this study.

### Measures

#### Potential risk factors

Three sets of questionnaires in supporting information (S1 and S2 Files) were developed based on information and data from the literature review [16,17]. The first set of questionnaires recorded the sociodemographic and obstetric variables (i.e., age, educational level, marital status, employment status and monthly family income, and intentions of pregnancy and parity). The second set of questionnaires recorded antenatal morbidities (i.e., GDM, preeclampsia, headache, nausea, and vomiting). The third set of questionnaires consisted of postnatal conditions (i.e., mode of delivery, gestation, gender of infant, and NICU admission).

#### Perinatal depressive symptoms

A 10-item EPDS [38] was used to assess the intensity of perinatal depressive symptoms in the three waves. The EPDS was initially developed for use with postnatal women [38] and was later validated for use with antenatal women [39]. The items were rated on a 4-point Likert scale ranging from 0 to 3. A higher score indicated greater intensity of perinatal depressive symptoms. Previous studies using confirmatory factor analyses suggested 1-factor [40], 2-factor [41], and 3-factor [42] structures of the EPDS in different countries. A Chinese version with satisfactory psychometric properties was used among antenatal [43] and postnatal women [40]. A study reported satisfactory sensitivity and specificity for identifying women in the Chinese population with mild to moderate depressive symptoms if the EPDS scores were > 9 and severe depressive symptoms if the EPDS scores were >14 in the second trimester, third trimester and six weeks postpartum [12]. Hence, cut-off scores of > 9 and > 14 were adopted in the present study.

### Analyses

IBM SPSS Statistics 24.0 (IBM Corporation, Armonk, NY, USA) was used. An analysis of variance (ANOVA) with repeated measures was used to compare EPDS scores with the different time points [44]. We preformed Wilks’ lambda test to compare differences between the means of EPDS scores in the three waves [44]. The percentages of the mild to moderate depressive symptoms (EPDS scores > 9) and severe depressive symptoms (EPDS scores > 14) were compared across three times proportion across three times [45]. We computed the non-parametric Cochran’s Q test for differences between three sets of proportions [45]. Reliability tests were used to test the internal consistency of the EPDS. The full information maximum likelihood estimation method was used to manage missing data [46]. The SEM was performed to assess the fit of the hypothesized model with the data and parameter estimate using the Analysis of Moment Structures (AMOS) software (version 24.0) [47]. Before conducting the SEM analyses, study variables were tested for normality [35] using the cutoffs for skewness (absolute value ≥ 2) and kurtosis (absolute value ≥ 7) [48]. The normal distribution assumption was imbedded in SEM [35]. To test the proposed hypothetical model based on previous conceptual models [33,34], a three-step approach was used [35].

In the first step, a series of EFAs were conducted to test the factor structure of the EPDS using principal axis factoring (PAF), which was recommended for estimating accurate factor loadings and unique variances [49]. The analysis of data structure in PAF focused on shared variances that were unique to individual measurement [49,50]. Items with a factor loading of > 0.3 were considered acceptable values [51]. Bartlett’s test of sphericity [52] and Kaiser–Meyer–Olkin (KMO) measure of sampling adequacy [53] were performed to check the case-to-variable ratio for the analysis. For a good factor analysis, the result of the Bartlett’s test and the value of the KMO were recommended to be < 0.05 [52] and > 0.60 [53], respectively.

In the second step, the CFA was performed to validate the factor structure constructed in the prior EFA [47] using the AMOS 24.0 software. Modifications indices (MI) were considered to improve the goodness-of-fit of the model with the evidence of misspecification associated with pairing error terms [35]. To determine the suitability of the model, several fit indices were used: chi-square/degree of freedom (*χ*^*2*^/*df*), incremental fit index (IFI), Tucker-Lewis index (TLI), comparative fit index (CFI), and root mean square error of approximation (RMSEA) [47,54]. The cut-off criteria for fit index were as follows: (1) *χ*^*2*^/*df* ≤ 3, (2) IFI > 0.9, (3) TLI > 0.9, (4) CFI > 0.9, and (5) RMSEA < 0.08 [35,55–57]. The third step was implemented to examine the hypothesized SEM relationships among latent variables using maximum likelihood estimation [47] following the criteria for an acceptable fit. Standardized regression coefficients (i.e., *β* value) were used to estimate the strength of paths between two variables.

## Results

A total of 663 pregnant women were invited to join this study (Fig 2); of which, 538 women completed the first wave questionnaire (response rate: 81.1%), 462 women completed the second wave questionnaire, and 361 women completed all three waves, yielding an overall attrition rate of 18%. The attrition reasons were fatigue and lack of time. The demographic characteristics between attrition and retention were not significantly different according to the chi-square tests. Descriptive statistics of sociodemographic variables, obstetric variables, antenatal morbidities, and postnatal conditions in the three waves are shown in Table 1.

**Table 1:** Sociodemographic, obstetric characterises, antenatal morbidities, and postnatal conditions of study participants (N = 361).

		N	%
1^st^ Wave: Sociodemographic and obstetric variables			
Age M (SD)	Range: 18–42	28.95 (4.82)	
Education level	≤ Secondary	194	53.7
	> Secondary^¶^	167	46.3
Marital status	Unmarried	41	11.4
	Married^¶^	320	88.6
Employment status	Unemployed	92	25.5
	Employed^¶^	269	74.5
Monthly total income	≤ $ 10000	34	9.4
	> $ 10000^¶^	327	90.6
Parity M (SD)	Range: 1–4	1.41 (0.62)	
Pregnancy intention	Unplanned	78	21.6
	Planned^¶^	283	78.4
2^nd^ Wave: Antenatal morbidities			
Gestational diabetes		34	9.4
Preeclampsia		11	30.
Nausea/Vomiting		140	38.8
Headache		85	23.5
3^rd^ Wave: Postnatal conditions			
Mode of delivery	Assisted delivery/Caesarean section	124	34.3
	Spontaneous vaginal delivery^¶^	237	65.7
Gestation of infant	< 37 weeks	30	8.3
Gender of infant	Female	163	45.2
NICU admission		69	19.1

M (SD) = Mean (Standard deviation); NICU = Neonatal intensive care unit;

^¶^ = Reference group.

Table 2 reports the mean scores of the EPDS for the first, second, and third waves as 8.21, 4.14, and 4.88, respectively, and Wilks’s Lambda test showed significance in the mean differences (*p* < 0.0001). The prevalence rates in the three waves were 34.6%, 10%, and 13.3% for mild to moderate depressive symptoms, and the rates were 9.4%, 2.5%, and 1.4% for severe depressive symptoms. Cochran’s Q tests indicated significant differences between the three waves of perinatal depressive proportions (*p* < 0.0001).

**Table 2:** Comparisons of depressive symptoms in three waves (N = 361).

Depressive symptoms scores	Range	M (SD)	*P* value
EPDS1	0–26	8.21 (4.62)	<0.0001^#^
EPDS2	0–18	4.14 (4.02)	
EPDS3	0–29	4.88 (4.05)	
Wilks’ lambda = 0.55, *F* (2, 359) = 149, *Ƞ*^2^ *=* 0.45			
Mild to moderate depressive symptoms	**N**	**%**	
EPDS1 > 9	125	34.6	<0.0001^§^
EPDS2 > 9	36	10.0	
EPDS3 > 9	48	13.3	
Cochran’s Q test: *χ*^*2*^(2) *=* 95.85			
Severe depressive symptoms			
EPDS1 > 14	31	9.4	<0.0001^§^
EPDS2 > 14	9	2.5	
EPDS3 > 14	5	1.4	
Cochran’s Q test: *χ*^*2*^(2) *=* 39			

EPDS1 = Edinburgh Postnatal Depression Scale in 1^st^ wave; EPDS2 = Edinburgh Postnatal Depression Scale in 2^nd^ wave; EPDS3 = Edinburgh Postnatal Depression Scale in 3^rd^ wave; M (SD) = Mean (Standard deviation); F = F-test; *df* = degree of freedom; *Ƞ*^2^ = Partial Eta Square; *χ*^*2*^ = Chi-square;

^#^ = Wilks’ lambda test;

^§^ = Cochran’s Q test.

Table 3 presents the values of skewness (0.78–1.29) and kurtosis (0.59–2.82), which indicated a normal distribution of the EPDS scores. Hence, the normal distributed data was fulfilled the SEM assumption before conducting our SEM analyses.

**Table 3:** Factor loadings, normality test and internal consistency for the Edinburgh Postnatal Depression Scale in three waves (N = 361).

Factor loadings	EPDS1		EPDS2		EPDS3	
	10-item	8-item	10-item	8-item	10-item	8-item
Item 1	0.35	0.33	0.36	0.34	0.37	0.33
Item 2	0.43	-	0.27	-	0.27	-
Item 3	0.27	-	0.43	-	0.55	-
Item 4	0.59	0.59	0.69	0.67	0.63	0.61
Item 5	0.63	0.63	0.76	0.77	0.56	0.56
Item 6	0.56	0.55	0.41	0.40	0.42	0.42
Item 7	0.66	0.67	0.67	0.70	0.64	0.66
Item 8	0.85	0.87	0.70	0.70	0.81	0.84
Item 9	0.68	0.68	0.71	0.71	0.65	0.66
Item 10	0.47	0.45	0.34	0.35	0.42	0.42
Eigenvalues	3.27	3.02	3.17	2.91	3.06	2.70
% of Variance	32.67%	37.76%	31.70%	36.38%	30.59%	33.76%
KMO	0.85	0.84	0.85	0.86	0.84	0.83
Bartlett’s test of sphericity						
*χ*^*2*^	978.42	847.61	929.63	779.38	919.30	724.51
*df*	45	28	45	28	45	28
*P*	< 0.0001	< 0.0001	< 0.0001	<0.0001	< 0.0001	<0.0001
Skewness	0.78	0.78	1.18	1.29	1.12	1.18
Kurtosis	0.76	0.59	1.25	1.69	2.81	2.82
α	0.81	0.81	0.80	0.80	0.79	0.78

EPDS1 = Edinburgh Postnatal Depression Scale in 1^st^ wave; EPDS2 = Edinburgh Postnatal Depression Scale in 2^nd^ wave; EPDS3 = Edinburgh Postnatal Depression Scale in 3^rd^ wave; *χ*^*2*^ *=* Chi-square; *df =* Degree of freedom; α = Cronbach’s alpha; KMO = Kaiser-Meyer-Olkin measure of sampling adequacy.

In the first step, a series of EFA tests was examined including 1, 2, and 3-factor structures using the PAF extraction procedures [49]. A scree plot with an elbow after the first eigenvalue indicates a well-fitting 1-factor model. Table 3 shows that the factor loadings of item 2, “look forward with enjoyment to things”, and item 3, “self-blamed necessarily when things went wrong”, were < 0.3, indicating low communalities. We examined the meaning of these two items, and found that the participants from the Macau region would interpret these two items differently because of the difference in tonal structures between Cantonese and Mandarin [58]. Such difference explains the different interpretation because the original Chinese version of the EPDS was validated among Mandarin speakers [43] and the majority of our participants are Cantonese speakers. Therefore, we decided to extract these two items in the SEM.

All factor loadings of the 8-item EPDS were > 0.3, and the percentage of explained total variance of the 8-item EPDS was higher than the 10-item EPDS, as shown in Table 3. Thus, the 8-item EPDS was considered sufficient for a coherent construct and it was used in steps 2 and 3. The KMO coefficients were 0.83–0.86, which suggested that these data were suitable for factor analysis [53]. Bartlett’s tests supported the factorability of the correlation matrix with a satisfactory result (*χ*^*2*^: 724.51–978.42, *p* < 0.001) [52]. The internal consistencies of the EPDS were satisfactory (0.78–0.82).

In the second step, three CFAs were performed to confirm an acceptable fit of a 1-factor structure for the 8-item EPDS in the three waves. The fit indices did not reach an acceptable benchmark in the initial CFA model for wave 1 and 3 because of model misspecification [47]. The MI captured the substantially larger value of an error covariance [47]. Thus, re-specification of the models was attempted to achieve a better fit of the model by correlating error terms 4 and 5 according to empirical rationales [47]. The modified CFA models of the EPDS1 and EPDS3 showed satisfactory fit indices (χ^2^/*df*: 1.74–2.06, IFI: 0.97–0.98, TLI: 0.96–0.98, CFI: 0.97–0.98, and RMSEA: 0.05) after model re-specification, as shown in Table 4. In the third step, the hypothesized SEM was tested to examine the relationships among study variables.

**Table 4:** Model fit statistics of study variables and structural equation model (N = 361).

	Model goodness-of-fit indices						
	*χ*^*2*^*/df*	IFI	TLI	CFI	RMSEA	LO90	HI90
Perinatal depressive symptoms							
EPDS1							
Initial model	4.35	0.92	0.89	0.92	0.10	0.08	0.12
Modified model	1.74	0.98	0.98	0.98	0.05	0.02	0.07
EPDS2							
Initial model	2.25	0.97	0.95	0.97	0.06	0.04	0.08
EPDS3							
Initial model	3.82	0.92	0.89	0.92	0.09	0.07	0.11
Modified model	2.06	0.97	0.96	0.97	0.05	0.03	0.08
Structural equation model	1.42	0.91	0.90	0.91	0.04	0.03	0.04

*χ*^*2*^/*df* = Chi = square / Degree of Freedom; IFI = Incremental Fit Index; TLI = Tucker-Lewis Index; CFI = Comparative Fit Index; RMSEA = Root Means Square Error of Approximation; LO90 = Lower limit of the 90% confidence interval of the index; HI90 = Upper limit of the 90% confidence interval of the index; EPDS1 = Edinburgh Postnatal Depression Scale in 1^st^ wave; EPDS2 = Edinburgh Postnatal Depression Scale in 2^nd^ wave; EPDS3 = Edinburgh Postnatal Depression Scale in 3^rd^ wave.

**Fig 3:**
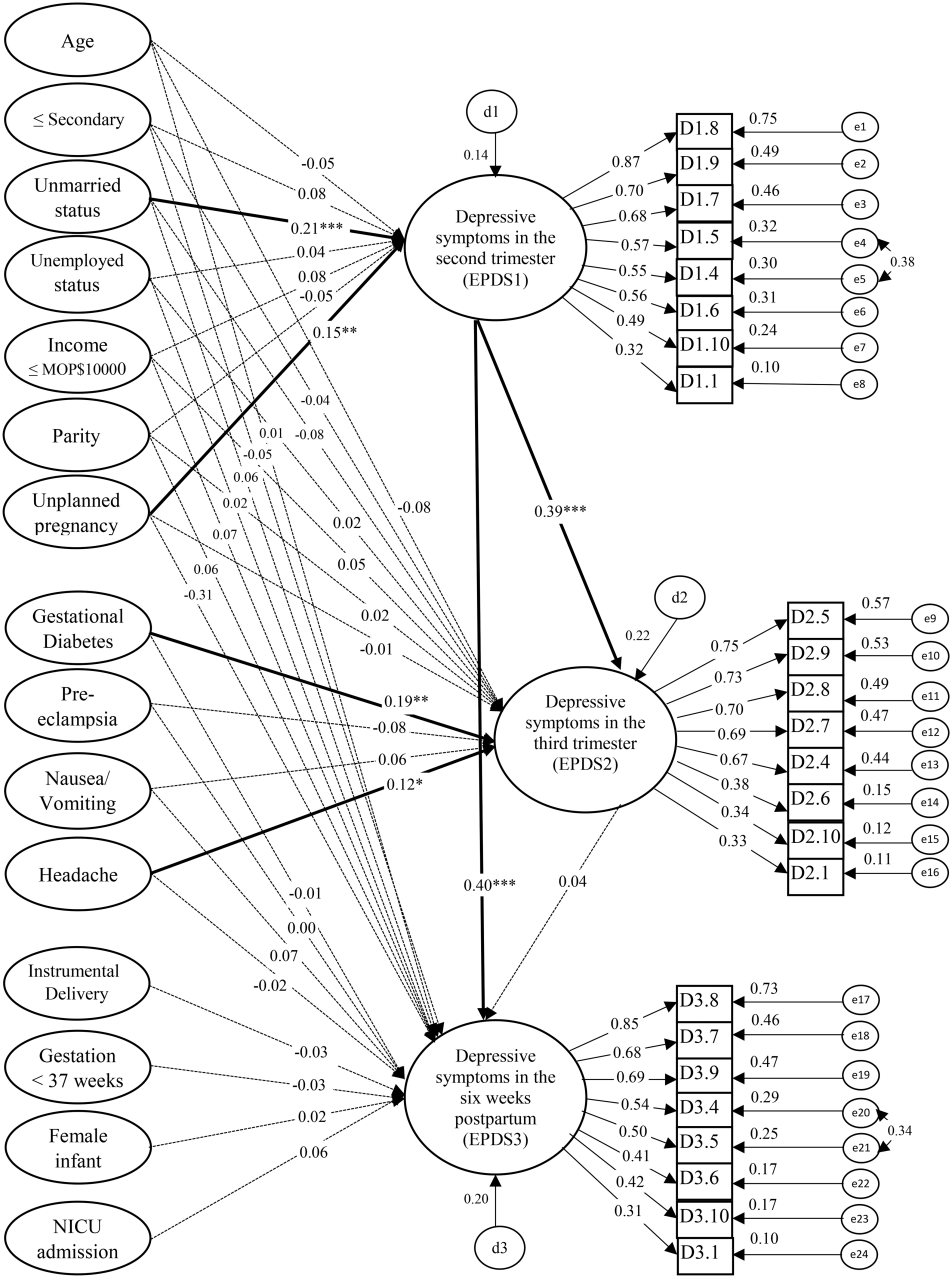
Results of the structural equation model of sociodemographic, obstetric factors, antenatal morbidities, and postnatal conditions on perinatal depressive symptoms in 3 waves (n = 361). NICU = Neonatal intensive care unit; EPDS1 = Edinburgh Postnatal Depression Scale in 1^st^ wave; EPDS2 = Edinburgh Postnatal Depression Scale in 2^nd^ wave; EPDS3 = Edinburgh Postnatal Depression Scale in 3^rd^ wave. Solid line indicates statistically significant finding, dotted line indicates non-significant finding, *e* error term, *d* residual term, ***p < 0.001, **p < 0.01,*p < 0.05.

As shown in Fig 3, unmarried status (*β =* 0.21, *p* < 0.0001) and unplanned pregnancy (*β =* 0.15, *p* < 0.001) were significantly associated with EPDS1. GDM (*β =* 0.18, *p* < 0.001) and headache (*β =* 0.14, *p* < 0.05) were significantly associated with EPDS2. EPDS1 was significantly associated with EPDS2 (*β =* 0.37, *p* < 0.001) and EPDS3 (*β =* 0.41, *p* < 0.0001). No significant relationships between postnatal conditions and EPDS3 were found. The SEM satisfactorily fitted the data (*χ*^*2*^/*df* = 1.42, IFI = 0.91, TLI = 0.90, CFI = 0.91, and RMSEA = 0.03) as presented in Table 4.

## Discussion

To the best of our knowledge, this paper presents the first three-wave prospective-longitudinal study in Macau which examined the pattern of perinatal depressive symptoms and relationships of study variables by using a SEM approach. Consistent with our previous psychometric study of the EPDS among Mandarin-speaking women in Mainland China [40], 1-factor structure of the EPDS were well-fitted among Cantonese-speaking women in Macau. However, factor loading of item 2 and item 3 of the EPDS were < 0.3 in this study. This may be because interpretation of two items were different between the language of Cantonese and Mandarin according to various tonal structures [58]. Given the different tone perception in Cantonese and Mandarin [59], two items were deleted prior to SEM analysis based on linguistic considerations. Thus, we used the 8-item EPDS rather than 10-item EPDS for our SEM approach.

Our findings indicated that the intensity of depressive symptoms was the highest in the second trimester compared with the third trimester and postnatal period. This finding contradicts the previous study [8] wherein the second trimester tends to be the calmest in terms of mood, even though the findings are similar to the Hong Kong study [12]. One possible interpretation could be the similarities between Hong Kong and Macau in terms of demographic characteristics, political backgrounds, geographic origins, behavioral patterns, and lifestyles [60]. Hence, the depressive pattern was similar in two places. Another possible reason could be the transition of parenthood during the second trimester, which might explain the escalating depressive symptoms [61]. The findings highlight the significance of monitoring for depressive symptoms in the second trimester.

In line with the previous evidence [14,15], antenatal depressive symptoms were significant risk factors for postnatal depressive symptoms. The present study showed that earlier antenatal depressive symptoms can predict later antenatal and postnatal depressive symptoms at six weeks postpartum. Surprisingly, antenatal depressive symptoms in the third trimester were not significantly associated with postnatal depressive symptoms, thus providing further support to the importance of early screening for depressive symptoms.

Unmarried mothers were more likely to have antenatal depressive symptoms, and this finding is consistent with the result of a previous study [18]. The increased risk of depressive symptoms among unmarried mothers may be linked to premarital sexual activities, which are considered unacceptable in most Asian countries [62] having a more conservative attitude towards sex. Being an unmarried mother is considered shameful or a stigma not only for herself, but also for her entire family [63]. In some instances, the family would reject and abandon unmarried mothers, which may create pressure and guilt, leading to depressive symptoms.

Consistent with the previous study [9], women with unplanned pregnancies have an increased risk for antenatal depressive symptoms. Unplanned pregnancy was associated with the late initiation of prenatal classes, and those who experience unplanned pregnancies were less likely to discuss the matters of pregnancy with their friends and relatives [64]. Moreover, one study showed that women with unplanned pregnancies have an increased risk for discontinuing mental health treatments after the confirmation of pregnancy compared with women with planned pregnancies [65]. Consequently, an unplanned pregnancy may contribute to the risk of antenatal depressive symptoms because of a lack of appropriate support and treatment.

Contrary to the previous study [23], a significant relationship was found between GDM and antenatal depressive symptoms. The relationship may be bidirectional and can be explained by psychosocial and physiologic mechanisms [66]. Antenatal depression is associated with glucocorticoid resistance [67], which may be related to GDM [66]. Depressed women experience difficulties in following complex GDM regimens, resulting in poor adherence [21]. However, whether the relationship between antenatal depressive symptoms and GDM is caused by neuroendocrine changes in depressive symptoms that trigger hyperglycemia or the effects of hyperglycemia cause depressive symptoms remains unclear [66]. Further investigation is necessary.

This study showed that headache was a significant factor of antenatal depressive symptoms, which is consistent with a previous study [25]. One possibility for this observation is that Chinese people tend to somatize their depressive symptoms as a physical symptom, such as headaches [26]. Another possibility is related to the neuropathology of serotoninergic and dopaminergic systems; an increased secretion of corticotrophin-releasing factor and changes in cortisol secretion might play a role in the relationship between depressive symptoms and headache [68].

Notably, postnatal conditions (mode of delivery, gestation and gender of infant and NICU admission) were not significantly related to postnatal depressive symptoms and these findings are contradictory with some previous studies [27,30,31]. This non-significant findings can be explained by multiple biological and environmental factors affecting depressive symptoms during postnatal period [69]. The genetic vulnerability had been found to be associated with postnatal depressive symptoms [70], especially in women who were exposed to environmental stresses during postnatal period [69]. Indeed, we do not know whether these four postnatal factors may not play important roles in postnatal depressive symptoms comparing to other possible factors (such as social support, parental competency, breastfeeding status or adverse postnatal complications). Thus, further investigations are warrant. Nevertheless, the present study showed that age, education, employment, income, parity, preeclampsia, nausea and vomiting, mode of delivery, gestation, gender of infant, and NICU admission did not influence perinatal depressive symptoms. Further elucidation is merited to investigate the directionality of the relationships.

Some limitations in this study should be highlighted. First, the high attrition rate may mean that the prevalence of perinatal depressive symptoms reported may underestimate or overestimate the actual prevalence. Second, non-randomized sampling in one hospital may limit the scope of generalization. Third, depressive symptoms were identified using a self-report screening tool, which is not equivalent to the gold standard of a structured diagnostic psychiatric interview. Finally, women who had a history of depression were not included, and thus determining whether the depressive episodes existed prior to the current pregnancy is impossible.

The findings of this study could help health care professionals identify high-risk women at different stages of the perinatal period and alert policy makers to the importance of ensuring adequate health resources to deal with perinatal depressive symptoms. Timely and tailor-made interventions could prevent adverse outcomes from the sequelae of perinatal depressive symptoms. Future studies are warranted to evaluate whether an improvement of GDM and headache could help to prevent perinatal depressive symptoms.

## Conclusion

Our results suggested that the second trimester may be a risky period to escalate depressive symptoms; thus, monitoring during the second trimester is vital. Early antenatal depressive symptoms were able to predict late antenatal and postnatal depressive symptoms. Unmarried status, unplanned pregnancy, GDM, and headache were risk factors for antenatal depressive symptoms. These results may help to develop target preventive interventions for perinatal women at risk. Further intervention studies are required to evaluate the effectiveness of the improvements of the target factors in preventing or improving perinatal depressive symptoms.

## Supporting information

S1 File Macau questionnaire (English version). (DOC)

S2 File Macau questionnaire (Chinese version). (DOC)

S3 File Data set for PLOS one (n = 361). (SAV)
