# Supplementary material for: Sociodemographic, obstetric characteristics, antenatal morbidities, and perinatal depressive symptoms: A three-wave prospective study
Source: PLoS One. 2018 Feb 8;13(2):e0188365. doi: 10.1371/journal.pone.0188365 (PMC5805167; doi:10.1371/journal.pone.0188365)
Supplement: S1 File — (DOC) [file pone.0188365.s001.doc]

**Macao English version of Questionnaires**

Please answer the follwing questions or tick “√” the answer:

| **Sociodemogrphic and obstetric data (1^st^ Wave)** | | | |
| --- | --- | --- | --- |
| 1 | Age: __________ |  |  |
| 2 | Educational Level: | □ ≤ Secondary | □ > Secondary |
| 3 | Marital status: | □ Umarried | □ Married |
| 4 | Empolyment Status: | □ Unemployment | □ Employment |
| 5 | Parity: ________ |  |  |
| 6 | Pregnancy intention | □ Planned | □ Unplanned |
| **Antenatal morbidities (2^nd^ Wave)** | | |  |
| 7 | Gestational Diabetes Mellitus | □ Yes | □ No |
| 8 | Gestational hypertension | □ Yes | □ No |
| 9 | Nausau and vomiting | □ Yes | □ No |
| 10 | Headache | □ Yes | □ No |
| **Postnatal condition (3^rd^ Wave)** | | |  |
| 11 | Modes of delivery: | □ Spontanous delivery | □ Assisted delivery  (Vaccum or Forceps) /  Cearean section |
| 12 | Gestation of infant | □ < 37 weeks | □ ≥ 37 weeks |
| 13 | Gender of infant | □ Male | □ Female |
| 14 | NICU admission | □ Yes | □ No |
| **Edinburg Postnatal Depression Scale (EPDS) (1^st^, 2^nd^ and 3^rd^ Waves)**  Please tick the answer, which comes closest to how you have felt ***IN THE PAST 7 DAYS.*** | | | |
| 1 | I have been able to laugh and see the funny side of things. | □ As much as I always could  □ Not quite so much now  □ Definitely not so much now  □ Not at all | |
| 2 | I have looked forward with enjoyment to things. | □ As much as I ever did  □ Rather less than I used to  □ Definitely less than I used to  □ Hardly at all | |
| 3 | I have blamed myself unnecessarily when things went wrong. | □ Yes, most of the time  □ Yes, some of the time  □ Not very often  □ No, never | |
| 4 | I have been anxious or worried for no good reason. | □ No, not at all  □ Hardly ever  □ Yes, sometimes  □ Yes, very often | |
| 5 | I have felt scared or panicky for not very good reason. | □ Yes, quite a lot  □ Yes, sometimes  □ No, not much  □ No, not at all | |
| 6 | Things have been getting on top of me. | □ Yes, most of the time I haven't been able to cope at all  □ Yes, sometimes I haven't been coping as well as usual  □ No, most of the time I have coped quite well  □ No, I have been coping as well as ever | |
| 7 | I have been so unhappy that I have had difficulty sleeping. | □ Yes, most of the time  □ Yes, sometimes  □ Not very often  □ No, not at all | |
| 8 | I have felt sad or miserable. | □ Yes, most of the time  □ Yes, quite often  □ Not very often  □ No, not at all | |
| 9 | I have been so unhappy that I have been crying. | □ Yes, most of the time  □ Yes, quite often  □ Only occasionally  □ No, never | |
| 10 | The thought of harming myself has occurred to me. | □ Yes, quite often  □ Sometimes  □ Hardly ever  □ Never | |
