# Supplementary material for: Sociodemographic, obstetric characteristics, antenatal morbidities, and perinatal depressive symptoms: A three-wave prospective study
Source: PLoS One. 2018 Feb 8;13(2):e0188365. doi: 10.1371/journal.pone.0188365 (PMC5805167; doi:10.1371/journal.pone.0188365)
Supplement: S2 File — (DOC) [file pone.0188365.s002.doc]

**澳門中文問卷**

**請在題目方格上加上 “√” 號**

| **個人資料及產科資料 (第一波資料)** | | | |
| --- | --- | --- | --- |
| 1 | 年齡： ＿＿＿＿ |  |  |
| 2 | 教育程度： | □ ≤ 中學程度 | □ > 中學程度 |
| 3 | 婚姻狀況： | □ 未婚 | □ 結婚 |
| 4 | 工作狀況： | □ 失業 | □ 全職工作 |
| 5 | 生產次數： ＿＿＿ |  |  |
| 6 | 懷孕計劃： | □ 計劃懷孕 | □ 意外懷孕 |
| **懷孕併發症 (第二波資料)** | | | |
| 7 | 懷孕期糖尿病: | □ 是 | □ 否 |
| 8 | 懷孕期血壓高： | □ 是 | □ 否 |
| 9 | 懷孕期嘔吐： | □ 是 | □ 否 |
| 10 | 頭痛： | □ 是 | □ 否 |
| **產後狀況 (第三波資料)** | | |  |
| 11 | 生產方式: | □ 自然分娩 | □助產生產(包括產鉗或真空吸引術) /剖腹生產 |
| 12 | 嬰兒週數: | □ < 37週 | □ ≥ 37週 |
| 13 | 嬰兒性別 | □ 男 | □ 女 |
| 14 | 特別護理嬰兒單位觀察 | □ 是 | □ 否 |
| **愛丁堡產後憂鬱症評估量表 (EPDS) (第一、二及三波資料)**  請選擇以下十題問題，每條題目都有四個答案，從四個答案中選出*一個*最能反映您過去七天感受的答案。 | | | |
| 1 | 我能看到事物有趣的一面，並笑得開心。 | □ 同以前一樣  □ 沒有以前那麼多  □ 肯定比以前少  □ 完全不能 | |
| 2 | 我欣然期待未來的一切。 | □ 同以前一樣  □ 沒有以前那麼多  □ 肯定比以前少  □ 完全不能 | |
| 3 | 當事情出錯時，我會不必要地責備自己。 | □ 大部分時候這樣  □ 有時候這樣  □ 不經常這樣  □ 沒有這樣 | |
| 4 | 我無緣無故感到焦慮和擔心。 | □ 一點也沒有  □ 極少有  □ 有時候這樣  □ 經常這樣 | |
| 5 | 我無緣無故感到害怕和驚慌。 | □ 相當多時候這樣  □ 有時候這樣  □ 不經常這樣  □ 一點也沒有 | |
| 6 | 很多事情衝著我而來，使我透不過氣。 | □ 大多數時候我都不能應付  □ 有時候我不能像平時應付得好  □ 大部分時候我都能像平時那樣應付得好  □ 我一直都能應付得好 | |
| 7 | 我很不開心，以致失眠。 | □ 大部分時候這樣  □ 有時候這樣  □ 不經常這樣  □ 一點也沒有 | |
| 8 | 我感到難過和悲傷。 | □ 大部分時候這樣  □ 相當時候這樣  □ 不經常這樣  □ 一點也沒有 | |
| 9 | **我不開心到哭。** | **□ 大部分時候這樣**  **□ 有時候這樣**  **□ 只是間中這樣**  **□ 沒有這樣** | |
| 10 | **我想過要傷害自己。** | **□ 相當多時候這樣**  **□ 有時候這樣**  **□ 很少這樣**  **□ 沒有這樣** | |
